# Receptor-like kinase complexes in plant innate immunity

**DOI:** 10.3389/fpls.2012.00209

**Published:** 2012-08-24

**Authors:** Christiaan Greeff, Milena Roux, John Mundy, Morten Petersen

**Affiliations:** Department of Biology, Copenhagen UniversityCopenhagen, Denmark

**Keywords:** receptor-like kinases, complexes, plant immunity, signaling, defense

## Abstract

Receptor-like kinases (RLKs) are surface localized, transmembrane receptors comprising a large family of well-studied kinases. RLKs signal through their transmembrane and juxtamembrane domains with the aid of various interacting partners and downstream components. The N-terminal extracellular domain defines ligand specificity, and RLK families are sub-classed according to this domain. The most studied of these subfamilies include those with (1) leucine-rich repeat (LRR) domains, (2) LysM domains (LYM), and (3) the *Catharanthus roseus* RLK1-like (CrRLK1L) domain. These proteins recognize distinct ligands of microbial origin or ligands derived from intracellular protein/carbohydrate signals. For example, the pattern-recognition receptor (PRR) AtFLS2 recognizes flg22 from flagellin, and the PRR AtEFR recognizes elf18 from elongation factor (EF-Tu). Upon binding of their cognate ligands, the aforementioned RLKs activate generic immune responses termed pattern-triggered immunity (PTI). RLKs can form complexes with other family members and engage a variety of intracellular signaling components and regulatory pathways upon stimulation. This review focuses on interesting new data about how these receptors form protein complexes to exert their function.

## INTRODUCTION

Autotrophs, like plants, are the source of nutrients for heterotrophs. Plants are members of complex communities and have co-evolved commensal and pathological relationships with microbes. A fine balancing act is required to effectively combat invasion by pathogenic heterotrophs while effectively guarding resources for vegetative and reproductive growth (King and Roughgarden, 1982). This entails appropriately timed activation of defense responses to conserve energy for producing numerous healthy progeny, thus increasing evolutionary fitness through this adaptive plasticity (Sultan, 2000). Detecting harmful heterotrophs and converting this recognition to intracellular signals aimed at combating the intruder and alerting surrounding tissue, is a major challenge, especially since pathogens co-evolve with their hosts to elude discovery (Frank, 1992; Lehti-Shiu et al., 2009).

Plant genomes encode a large number of surface receptor-like kinases (RLKs) to perceive different signals from both distal cells responding to stresses such as herbivore feeding or to the presence of pathogens through detection of non-self molecules (Shiu and Bleecker, 2001). RLKs generally have an extracellular ligand-binding domain, a membrane-spanning region, a juxtamembrane (JM) domain, and a serine/threonine kinase domain. Equivalent mammalian receptors from the Pelle/IRAK family differ in usually employing a cytosolic tyrosine kinase domain (Gish and Clark, 2011). A conserved aspartate in the catalytic loop of most kinases is required for catalytic activity. Ser/Thr kinases mostly have an arginine preceding this catalytic aspartate. Kinases with such residues are termed RD kinases, although most RLKs implicated in microbial detection are non-RD kinases, lacking an arginine preceding the catalytic aspartate. They in general require additional proteins to modulate their function (Johnson et al., 1996; Dardick and Ronald, 2006). An important example is BAK1, which interacts with many Arabidopsis RLKs, and is required for their activity (discussed below).

The plant RLK family has more than 600 members in *Arabidopsis* (Shiu et al., 2004). RLKs are divided into 44 sub-families depending on their N-terminal domains. While RLKs have been implicated in many biologically important processes (Gish and Clark, 2011), this review focuses on RLKs involved in pathogen detection.

**FIGURE 1:**
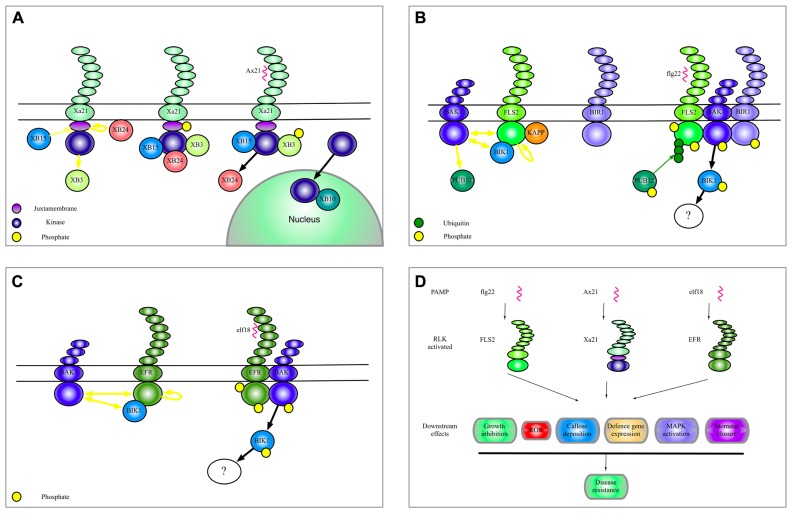
**Complexes of Xa21, FLS2, and EFR.** **(A)** A model to summarize the current data regarding Xa21 function as discussed in this manuscript. **(B)** Illustration of the complexes formed by the RLK-FLS2. The yellow dots indicate phosphorylation of a protein. Yellow arrows indicate phosphorylation of a substrate protein. Yellow blunt arrows indicate dephosphorylation of a substrate protein. Green dots and green arrows indicate ubiquitination. Black arrows indicate translocation, association or dissociation. **(C)** Selected interactors of the RLK EFR. **(D)** Shows biological effects of selected RLK activation.

Plant RLKs involved in immunity are so-called pattern-recognition receptors (PRRs) that detect pathogen-associated molecular patterns (PAMPs) and, upon binding of their cognate elicitors, initiate a well-characterized set of defense responses termed PAMP-triggered immunity (PTI). Features of PTI include reactive oxygen species (ROS) production, callose deposition, generation of secondary messengers, and defense gene expression (Jones and Dangl, 2006). RLK elicitation also leads to activation of several mitogen-activated protein (MAP) kinases (Suarez-Rodriguez et al., 2007; Mithoe et al., 2011). PAMPs, and more broadly, microbial-associated molecular patterns (MAMPs) and damage-associated molecular patterns (DAMPs), can activate RLKs (Lerouge et al., 1990; Gómez-Gómez and Boller, 2000; Zipfel et al., 2006; Krol et al., 2010). Binding of PAMPS and DAMPS to their specific receptors leads to a broad range of downstream signaling events and effects. Figures 1A–C gives an overview of some of the complexes of Xa21, FlS2, and EF-Tu receptor (EFR) that will be discussed in this review. **Figure 1D** shows biological effects of FLS2, Xa21 and EFR.

## THE LRR FAMILY

The best-studied members of the leucine-rich repeat (LRR)-RLK family are the non-RD kinases AtFLS2, AtEFR, and OsXa21 (Park et al., 2010a) and the RD kinase BAK1 (Chinchilla et al., 2007; Heese et al., 2007). These receptors present the core of our current knowledge regarding RLKs involved in defense.

## Xa21

The Xa21 extracellular domain is composed of 23 LRRs and was one of the first eukaryotic RLKs found to be involved in resistance (Song et al., 1995; Wang et al., 2006). Xa21 binds the *Xanthomonas oryzae* pv. *oryzae* (Xoo) secreted tyrosine (Tyr) *O*-sulfonation peptide AxY^S^22 (Lee et al., 2009). Much has been learned about the function of Xa21. For example, the amino acids Ser686, Thr688, and Ser689 in the cytosolic JM domain are important for stability and for endoplasmic reticulum (ER) processing (Xu et al., 2006; Park et al., 2010a). Phosphorylation of residues in the JM domain is also critical for the activation of Xa21 and binding of at least four Xa21-binding proteins named XB3, XB15, XB24, and XB10 (OsWRKY62; Park et al., 2010b) associated with Xa21 via the JM domain. These interactions are all dependent on Thr705 since mutation of this JM domain residue abolishes XB-Xa21 binding (Chen et al., 2010a).

XB3 is an E3 ligase important for Xa21 accumulation and is a substrate for Xa21 kinase activity, although the biological relevance of this relationship is still unclear. After Xa21 binds AxY^S^22, XB3 is activated by transphosphorylation and likely leads to cleavage of a negative regulator of defense or even of itself, allowing other interactions to take place (Wang et al., 2006).

Xa21 is regulated by two proteins through phosphorylation; XB15, a protein phosphatase 2C (PP2C) and XB24, a protein with intrinsic ATPase activity (Park et al., 2008). XB15 dephosphorylates Xa21 and XB15 over-expression reduces *Xoo* resistance while *xb15* null-mutants exhibit increased cell death and resistance to *Xoo*. This would point to a negative regulatory role of XB15. On the other hand, XB24 promotes autophosphorylation of Xa21 and may be required to prevent proteolytic cleavage of Xa21. The complex between XB24 and Xa21 dissociates upon *Xoo* infection or AxY^S^22 binding (Chen et al., 2010b). Phosphorylation, especially in the JM domain, plays a critical role in Xa21 stability. It is clear that autophosphorylation of certain residues in Xa21 promotes an inactive state but the exact changes in phosphorylation status upon pathogen infection remain largely unknown.

Xa21 binds to the WRKY transcription factor XB10 and this binding requires an active Xa21 kinase domain. Binding of the AxY^S^22 peptide to Xa21 leads to translocation of a Xa21 kinase domain-GFP fragment to the nucleus where it interacts with XB10. The nuclear translocation is important for *Xoo* resistance and the Xa21 kinase domain/XB10 complex probably affects defense gene expression (Park and Ronald, 2012). Whether this or a similar mechanism also applies to other RLKs is currently unknown, but future studies will likely address this issue. Recently, a large-scale yeast two-hybrid study revealed yet another set of Xa21 interacting partners (Seo et al., 2011). Although the biological significance of these discoveries in signaling remains to be seen, they may provide interesting clues to the functions of Xa21 and other RLKs.

To help proteins fold properly, the ER contains a number of chaperones including BiPs (binding immunoglobulin protein) that bind N-glycosylated proteins and direct them to the ER (Molinari and Helenius, 2000). Xa21 is also N-glycosylated and interacts with BiP3, an HSP70-like ATPase located in the ER, and this is important for correct folding and functioning of the protein (Park et al., 2010a). While a pool of Xa21 locates to the PM where AxYS22 ligand is perceived, the majority of the receptor is found in the ER.

## AtFLS2

The FLS2 (flagellin sensing 2) receptor recognizes the well-conserved protein flagellin from a broad class of bacterial plant pathogens including *Pseudomonas syringae* pv. *tomato* (*Pto*) DC3000 (Gómez-Gómez and Boller, 2000). Direct binding of the flagellin-derived peptide flg22 has been shown using ^125^I-labeled peptides (Chinchilla et al., 2006), but a recent report also implicates FLS2 in unsulfonated *Xoo* Ax21 peptide perception. These two peptides are not sequence related, which makes the finding quite astonishing (Danna et al., 2011).

FLS2 was recently shown to form homo-dimers independently of flg22 binding, but whether this dimerization is important for receptor function is not known (Sun et al., 2012). However, it is well-established that FLS2 forms heterodimers with BRI1-associated kinase 1 (BAK1) (Chinchilla et al., 2007; Schulze et al., 2010) in the presence of bound flg22. BAK1 is a common component in many RLK signaling complexes, and was first identified for its requirement in brassinosteroid signaling via the receptor BRI1 (Li et al., 2002). The essential role of BAK1 in flg22 sensing was revealed by the marked reduction of flg22-induced responses in *bak1* plants (Chinchilla et al., 2007; Heese et al., 2007). Importantly, the BAK1–FLS2 interaction most likely does not compete with other known BAK1 interactors such as BRI1, and the BAK1–FLS2 interaction is therefore not responsible for BR-mediated PAMP defense suppression (Albrecht et al., 2012). BAK1 is a member of the somatic embryogenesis receptor kinase (SERK) family comprising 5 members, SERK1, SERK2, BAK1/SERK3, BAK1-like (BKK1)/SERK4, and SERK5. FLS2 interactions with SERK1, SERK2, SERK5, and BKK have been detected, but its predominant association is with BAK1. BAK1 and BKK1 are thought to act cooperatively in PAMP signaling and resistance to biotrophic pathogens (Roux et al., 2011).

BAK1 and FLS2 also interact with Botrytis-induced kinase 1 (BIK1), which is a receptor-like cytoplasmic kinase (RLCK) implicated in resistance to necrotrophic pathogens (Veronese et al., 2006). BAK1 and FLS2 phosphorylate BIK1 (Lu et al., 2010) and BIK1 in turn phosphorylates both FLS2 and BAK1. This is thought to be an important signal amplification mechanism. However, since FLS2 has been shown to have very low catalytic activity *in vitro* (Schwessinger et al., 2011), BAK1 probably possesses the predominant kinase activity influencing BIK1 phosphorylation. The BIK1–FLS2/BAK1 association is decreased after flg22 sensing, suggesting that BIK1 is released to activate downstream signaling components (Lu et al., 2010). BIK1’s role in PTI is dependent on complex interactions with major immune response regulators and may thus provide RLK signaling complexes with the ability to discriminate between biotrophic and necrotrophic pathogens (Laluk et al., 2011). Importantly, *bik1* mutants display enhanced susceptibility to *Pto* DC3000, reduced flg22 responsiveness, as well as compromised flg22-induced resistance to virulent *Pto *DC3000. The BIK1-related kinases, PBS-like kinase 1 (PBL1) and PBL2 also interact with FLS2 and BAK1. *pbl1* mutants show less reduction in PTI responses but the effect seems to be additive to BIK1 function (Zhang et al., 2010).

BAK1, BKK1, SERK1, and SERK2 have also been shown to interact with BIR1 (BAK1-interacting receptor-like kinase 1), an active protein kinase. The *bir1* mutant exhibits increased resistance to biotrophic *Pto* DC3000 and *Hyaloperonospora* *arabidopsidis* Noco2, due to apparent *R* protein activation (Wang et al., 2011). The *bir1 *phenotype is partially rescued in *bir1* *pad4 *double mutants, and is completely rescued in the *bir1 pad4 sobir *(*suppressor of bir1-1*) triple mutant. Phytoalexin deficient 4 (PAD4) is one of the critical components required for Toll/interleukin-1 receptor (TIR) R protein signaling. Many constitutively active defense phenotypes that result from activated TIR R proteins are suppressed by PAD4 loss of function (Wiermer et al., 2005; Palma et al., 2010; Zhang et al., 2012). The aforementioned results thus indicate that the *bir1* phenotype is partly dependent upon R protein activation, although the majority of defense induction in *bir1* occurs through SOBIR1. SOBIR1 is also a RLK, and over-expression of SOBIR1 leads to activation of cell death. SOBIR1 does not function in flg22 sensing and does not interact with BIR1. Exactly how loss of BIR1 activates SOBIR1 is a mystery (Gao et al., 2009), and it is still uncertain whether BIR1 has a role in the PAMP signaling pathway.

Kinase-associated protein phosphatase (KAPP) interacts with the FLS2 kinase domain (Gómez-Gómez and Boller, 2000), and this interaction may be important for receptor endocytosis upon activation as was found for AtSERK1 (Shah et al., 2002). KAPP has also been found in complexes with other RLKs (Williams et al., 1997; Stone et al., 1998) but whether it functions as a general regulator of a broader spectrum of RLKs needs to be explored.

FLS2 also interacts with E3 ligases that polyubiquitinate the receptor after flg22 signaling. FLS2 is subsequently degraded by the proteasome, which might constitute a mechanism for attenuation as has been described for the mammalian Toll-like receptor 4 (TLR4) and TLR9 (Chuang and Ulevitch, 2004). Plant U-Box 12 (PUB12) and PUB13, both E3 ubiquitin ligases, have been shown to be BAK1 phosphorylation targets, and this modification is required for their association with FLS2. This phosphorylation is reminiscent of the previously mentioned Xa21 phosphorylation of XB3. PUB12 and PUB13 control flg22-dependent, proteasome-mediated degradation of FLS2 (Lu et al., 2011), making this system important for FLS2 signaling attenuation, together with receptor endocytosis (Salomon and Robatzek, 2006).

Despite being a transmembrane protein, FLS2 does not depend critically on N-glycosylation for its function as has been found for EFR (Nekrasov et al., 2009; Saijo et al., 2009; Häweker et al., 2010). However, FLS2 has recently been shown to interact with the reticulon-like proteins RTNLB1 and RTNLB2. RTNLB1/2 are together involved in regulating FLS2 transport from the ER to the plasma membrane (Lee et al., 2011). In addition, stomatal cytokinesis defective 1 (SCD1) was identified by mass spectrometry as an FLS2 interaction partner. *Scd1* mutants display SA-dependent enhanced resistance to infection with *Pto* DC3000, as well as enhanced accumulation of *PR1* transcripts and hydrogen peroxide. However, the same mutants are less sensitive to PAMPs, with reduced seedling growth inhibition and ROS production in response to flg22 or elf18 (Korasick et al., 2010).

## EF-Tu RECEPTOR

EF-Tu receptor is a LRR-RLK that recognizes the peptide elf18 from bacterial elongation factor (EF)-Tu. EFR and BAK1 have also been shown to interact in a ligand-dependent manner (Roux et al., 2011). Indeed, many of the signaling components downstream of EFR and FLS2 are shared. While both EFR and FLS2 are capable of associating with all members of the SERK family, BKK1, SERK1, SERK2 have a stronger association with EFR than with FLS2 (Roux et al., 2011). This might allow EFR to avoid pathogen effector action on the single SERKs. Studies of SERK function have been difficult due to their apparent redundancy and the lethality of some double mutants such as *serk1 serk2* and *bak1-4 bkk1-1 *(Colcombet et al., 2005; He et al., 2007). However, the discovery of a novel allele of *bak1*, *bak1-5*, enabled study of non-lethal *bak1-5 bkk1* double mutants. This revealed that BAK1 and BKK1 act cooperatively in PAMP signaling (Roux et al., 2011; Schwessinger et al., 2011).

BIK1 is phosphorylated upon elf18 and flg22 treatment (Lu et al., 2010). Given the many parallels between FLS2 and EFR, it is possible that transphosphorylation of the EFR/BAK1 complex also occurs, although direct proof is still lacking. In contrast to FLS2, but similarly to Xa21, N-glycosylation is critical for EFR function and EFR is subject to ER quality control that requires several chaperones involved in ER-QC for full activity (Häweker et al., 2010).

## PEPR1

In contrast to the three receptors described above, Pep1 receptor 1 (PEPR1) binds AtPep1 (Yamaguchi et al., 2006) a DAMP derived from the precursor gene *PROPEP1*. PEPR1 and PEPR2 act redundantly to perceive AtPep1. BAK1 was shown to interact with PEPR1 like FLS2 and EFR (Postel et al., 2010). PEPR1 possesses a putative guanylyl cyclase (GC) domain and cGMP production by the purified RLK was shown *in vitro* (Qi et al., 2010). Interestingly, a GC domain is also present in BRI1 and was shown to have a catalytic function *in vitro* (Kwezi et al., 2007). This cGMP generated after elicitation may trigger a cyclic nucleotide-activated Ca^2^^+^ channel as part of its signaling activity (Ali et al., 2007).

## LysM FAMILY

Chitin elicitor receptor kinase 1 (CERK1) is the best studied Arabidopsis LysM-RLK (Kaku et al., 2006; Miya et al., 2007; Wan et al., 2008), and direct binding of chitin to CERK1 has been detected (Iizasa et al., 2010; Petutschnig et al., 2010). Unlike FLS2 and EFR, CERK1’s perception of fungal chitin is BAK1-independent. In rice, Chitin elicitor-binding protein (CeBIP), a LysM domain-containing protein, associates with OsCerk1 and these proteins function together in a hetero-oligomer receptor complex to elicit chitin signaling in a ligand-dependent manner (Shimizu et al., 2010). Two LysM domain proteins, LYM1 and LYM3, have recently been shown to be important for peptidoglycan (PGN), but not chitin recognition. LYM1 and LYM3 are not functionally redundant, and it has been proposed that LYM1, LYM3 and CERK1 may form a complex or complexes. *cerk1* is hypersusceptible to *Pto* DC3000 and shows reduced sensitivity to PGN, phenocopying *lym1/lym3*, however CERK1 does not bind to PGN. Further, given the fact that neither LYM1 nor LYM3 contain a cytoplasmic domain, a LYM1/LYM3/CERK1 complex seems likely (Willmann et al., 2011). RLKs often hetero-oligomerize for optimal functioning as seen in the co-operativity of FLS2/BAK1, EFR/BAK1 and PEPR1/PEPR2.

## CrRLK1L FAMILY

Another RLK, FERONIA (FER) was first shown to control pollen tube reception (Escobar-Restrepo et al., 2007). However, the expression of FER throughout the plant suggests a general function not strictly associated with root development or pollen tube reception. Indeed, FER has more recently been shown to aid powdery mildew (PM) penetration into host cells (Kessler et al., 2010) and to be responsible for susceptibility to the oomycete *H. arabidopsidis* (Nibau and Cheung, 2011). It is suspected that FER might play a role in controlling localization of MLO family proteins, known to be important for PM infection (Consonni et al., 2006), as it does for NTA during pollen tube reception. This however still needs to be shown, as well as whether ROS signaling has an effect on MLO localization. Given the many roles of FER it is not surprising to find that it is important for disease resistance as well.

FER appears to exert is signaling functions by controlling ROS production. FER was shown to interact with guanine nucleotide exchange factors (GEFs) that regulate RHO GTPases (RAC/ROPs). RAC/ROP is known play important roles in stress-induced responses. In rice, the binding of a RAC/ROP called Rac GTPase to NADPH oxidases has been characterized, and Rac GTPase was show to be required for PAMP-mediated ROS production (Wong et al., 2007). In *Arabidopsis*, Rop2 was shown to co-immunoprecipitate with FER. In addition, transgenic plants expressing constitutively active, GTP-bound Rop2 displayed increased ROS production (Cheung and Wu, 2011). This indicates that a FER-GEF-RAC/ROP complex is likely able to effect ROS production. While ROS play a role in root development, there are hints that FER is involved in ROS production during PAMP signaling in leaves. For example, FER is enriched in detergent-resistant membranes (DRMs) after flg22 treatment, and FER shows flg22-induced phosphorylation (Benschop et al., 2007). *Fer* mutants also exhibit enhanced ROS production, and aberrant stomatal responses upon flg22 treatment (Keinath et al., 2010). The increase in ROS production in the *fer* mutant is puzzling given the reduced Rho GTPase activity in this mutant (Duan et al., 2010). The relationship between FLS2 and FER in the control of ROS production is very interesting and should attract attention in the near future.

## CONCLUDING REMARKS

There have been enormous advancements in our knowledge about RLK signaling in the last decade, but many questions still remain unanswered. For example, the link between the PRR receptors and production of ROS and activation of MAP kinases is still missing. Nevertheless, a quite comprehensive picture of the route from receptor activation to enhanced defense gene expression has emerged for Xa21 and similar data for FLS2 and EFR are sure to come to light.

## Conflict of Interest Statement

The authors declare that the research was conducted in the absence of any commercial or financial relationships that could be construed as a potential conflict of interest.
